# Adaptive Evolution of Serotype O Foot‐and‐Mouth Disease Virus Under Vaccine Pressure: Combined VP1 T142/Q153 Mutations Drive Antigenic Alteration and Immune Evasion

**DOI:** 10.1155/tbed/4808637

**Published:** 2026-07-13

**Authors:** Nan Cao, Yamei Li, Xinghua Chen, Qiongqiong Zhao, Jinyan Zhang, Xianfei Shang, Junling Hou, Jinming Zhang, Bo Yin, Jinyan Wu, Xiangmin Li, Ping Qian

**Affiliations:** ^1^ National Key Laboratory of Agricultural Microbiology, Hubei Hongshan Laboratory, Huazhong Agricultural University, Wuhan, 430070, Hubei, China, hzau.edu.cn; ^2^ College of Veterinary Medicine, Huazhong Agricultural University, Wuhan, 430070, Hubei, China, hzau.edu.cn; ^3^ Key Laboratory of Preventive Veterinary Medicine in Hubei Province, The Cooperative Innovation Center for Sustainable Pig Production, Wuhan, 430070, Hubei, China; ^4^ State Key Laboratory for Animal Disease Control and Prevention, College of Veterinary Medicine, Lanzhou University, Lanzhou Veterinary Research Institute, Chinese Academy of Agricultural Sciences, Lanzhou, 730046, China, caas.cn; ^5^ Shanghai Shen Lian Biomedical Corporation, Shanghai, 200241, China; ^6^ Hubei Jiangxia Laboratory, Wuhan, 430200, China

**Keywords:** antigenicity, combined mutation, FMDV, immune evasion, VP1 G–H loop

## Abstract

Foot‐and‐mouth disease virus (FMDV) escapes host immune surveillance via adaptive evolution driven by vaccine‐mediated selective pressure, leading to persistent breakthrough infections in immunized animals. In this study, the dominant neutralizing epitope VP1 G–H loop (141–160 aa) was analyzed among 46 serotype O FMDV strains belonging to Southeast Asian (SEA) and Middle East–South Asian (ME‐SA) topotypes isolated during 1980–2019. The key amino acid residues at VP1 positions 142 and 153 underwent sequential stepwise evolution across three phases under natural selection. Based on the reverse genetic system of Wt, single‐site mutant (T142P, Q153P) and double‐site mutant (T142P&Q153P) strains were rescued. These mutation sites were further introduced into an efficient FMDV nanoparticle vaccine to construct four vaccine candidates. Mouse immunization verified all vaccines conferred solid protection against Wt and single‐mutant strains, yet protection efficacy was greatly impaired against the double mutant. The double‐mutant vaccine elicited high‐level neutralizing antibodies against the double‐mutant strain (T142P&Q153P) with a titer of 1:426.67, 10–20 folds higher than Wt (1:21.33), T142P (1:32), and Q153P (1:42.67) vaccines. Consistent results were also obtained in pigs immunized with commercial inactivated vaccines. Collectively, combined mutations at VP1 142 and 153 reshape viral antigenicity and act as core drivers of FMDV immune evasion. Integrating such immune escape hotspots into vaccine antigens can broaden neutralizing antibody coverage, offering an experimental basis for clarifying FMDV evolution and developing broad‐spectrum vaccines.

## 1. Introduction

Foot‐and‐mouth disease (FMD), caused by FMD virus (FMDV), is an acute, febrile, and highly contagious disease that primarily affects cloven‐hoofed animals such as cattle, pigs, and sheep. FMDV spreads rapidly with a high incidence rate, severely impairing the productivity of infected animals and causing high mortality in young livestock, thereby inflicting substantial economic losses on the livestock industry. Consequently, it is listed by the World Organization for Animal Health (OIE) as a priority notifiable infectious disease [1, 2]. As a member of the *Aphthovirus* genus within the *Picornaviridae* family, FMDV lacks a proofreading mechanism during replication, which facilitates the generation of viral quasispecies [2, 3]. This renders FMDV a dynamically evolving pathogen, constantly mutating and adapting to its environment, thereby developing resistance to host immune responses and antiviral therapies, ultimately leading to immune evasion. It has been confirmed that FMDV undergoes gradual but continuous sequence variation, with a genomic annual mutation rate of ~0.5%–1.0%. Driven by this high mutation rate and significant antigenic diversity, such rapid evolution frequently results in antigenic mismatches between circulating strains and existing vaccines, which is the primary cause of viral immune evasion [4–6].

FMDV features an icosahedral capsid composed of four structural proteins (VP1, VP2, VP3, and VP4), which encloses a single‐stranded positive‐sense RNA [4, 7]. Within the structural architecture of FMDV, the VP1 protein is a core molecule mediating viral infection, immune responses, and pathogenicity. Located on the surface of the virion, VP1 is the primary immunogenic protein and plays an irreplaceable role in key processes such as viral entry into host cells, antigenicity determination, and virulence regulation. VP1 exerts multidimensional regulatory effects during viral infection, involving the induction of neutralizing antibodies (nAbs), mediation of humoral and cellular immune responses, initiation of host cell death programs, and promotion of viral replication [8, 9]. Notably, VP1 further finely regulates infection dynamics through interactions with host proteins. For example, its binding to sorcin can significantly inhibit the production of type I interferons and disrupt the nuclear factor‐κB (NF‐κB) signaling pathway, ultimately creating favorable conditions for persistent viral infection [10]. In contrast, the VP1 protein can alleviate the ribosomal protein SA (RPSA)‐mediated inhibition of the mitogen‐activated protein kinase (MAPK) pathway, thereby promoting viral replication [11]. In addition, certain regions on the surface of the VP1 protein form antigenic epitopes, which can specifically bind to antibodies produced by the host immune system and serve as key sites for the virus to be recognized by the immune system. For instance, the G–H loop region of the VP1 protein (typically containing amino acids 141–160) is an important antigenic epitope, and the amino acid sequence and spatial structure of this region play a crucial role in the binding affinity between the virus and antibodies [12]. Structurally, the G–H loop of VP1 exhibits both conserved and variable characteristics. On the one hand, it contains a highly conserved RGD (arginine‐glycine‐aspartic acid) motif, which is essential for viral entry through binding to host integrin receptors—a diverse family consisting of 24 α‐β heterodimers, all of which can recognize the RGD sequence in their natural ligands [13, 14]. On the other hand, this loop contains linear B‐cell epitopes that can induce neutralizing antibodies and trigger strong protective immune responses, endowing intact virions with significant immunogenicity. Therefore, as a key immunodominant epitope of FMDV, the VP1 G–H loop can effectively mediate the body to generate specific protective immune responses and also serves as an important structural basis for maintaining the immunogenicity of vaccines and supporting immune protective efficacy [15]. This conclusion is also supported by research by Fernández‐Sainz et al. [12] when they replaced the hypervariable epitope in the VP1 G–H loop of an adenovirus‐vectored FMDV vaccine (Ad5‐FMD) with a heterologous epitope from porcine reproductive and respiratory syndrome (PRRS) virus, they found that the immune response and vaccine efficacy in pigs were significantly reduced, further highlighting the core status of the VP1 G–H loop epitope in protective immunity [12].

Notably, the G–H loop of VP1 also contains a hypervariable region (HV) that affects antibody binding and immune evasion. Although the receptor‐binding domain (RBD) at positions 145–147 is crucial for viral adsorption and receptor binding and remains conserved, other sequences within this loop are prone to mutation [16]. Since protein function is closely related to amino acid sequence, frequent amino acid mutations in VP1 during FMDV evolution can alter its spatial conformation, thereby leading to changes in viral antigenicity. This antigenic variation enables the virus to evade host immune recognition, representing a key mechanism underlying the reduced protective efficacy of vaccines and the increased difficulty in FMD prevention and control.

In this study, we analyzed the VP1 G–H loop (141–160 aa)—the most critical neutralizing epitope of 46 serotype O FMDV strains of the Southeast Asian (SEA) and Middle East–South Asian (ME‐SA) topotypes isolated between 1980 and 2019, with relevant data retrieved from the NCBI database. The results revealed that over time, VP1 underwent site‐specific mutations at residues 142 and 153, corresponding to the substitutions T142P and Q153P. To verify the impact of these mutations on immune evasion, we successfully rescued four FMDV mutant strains (Wt, T142P, Q153P, and T142P&Q153P) using reverse genetics based on the FMDV O/MYA/01/1998 strain, introducing mutations at residues 142 and 153 of VP1. To evaluate the antigenic changes induced by these mutations, we integrated the mutations into a previously developed high‐efficiency FMDV nanoparticle vaccine, constructing four corresponding nanoparticle vaccines. Humoral immune responses play a key role in overcoming FMDV infection, and systemic neutralizing antibodies are critical mediators for virus clearance [17]. We immunized mice with these four candidate vaccines and comprehensively analyzed the effects of the key VP1 mutations T142P and Q153P on antigenicity and immune evasion through cross microneutralization (MN) assays and in vivo cross‐protection tests.

The results demonstrated that all four nanoparticle vaccines provided strong protection against the Wt and single‐mutant strains, but their protective effect against the double‐mutant strain was significantly reduced. Notably, the neutralizing antibodies induced by the double‐mutant nanoparticle vaccine were significantly higher than those induced by the Wt and single‐mutant nanoparticle vaccines, indicating that it can elicit a higher level of broadly neutralizing antibodies with a stronger and more extensive protective efficacy. Subsequent evaluation of pigs immunized with different commercial inactivated vaccines confirmed a similar trend: all tested commercial vaccines effectively protected against the Wt and single‐mutant strains but showed a reduced protective effect against the double‐mutant strain. This study preliminarily explored and verified the feasibility of integrating immune evasion‐related hotspot mutations into VP1 to expand the activity spectrum of antigen‐induced antibodies. Additionally, the research indicated that the combined mutations at VP1 residues 142 and 153 promote FMDV escape from vaccine‐induced immunity and alter viral antigenicity. Our findings hold significant theoretical value and practical significance for revealing the pathogenic mechanism of FMDV, developing new vaccines, and formulating scientific and effective prevention and control strategies.

## 2. Results

### 2.1. Evolution and Adaptive Mutation of Key Amino Acid Sites in VP1 G–H Loop of Serotype O FMDV

**Figure 1:**
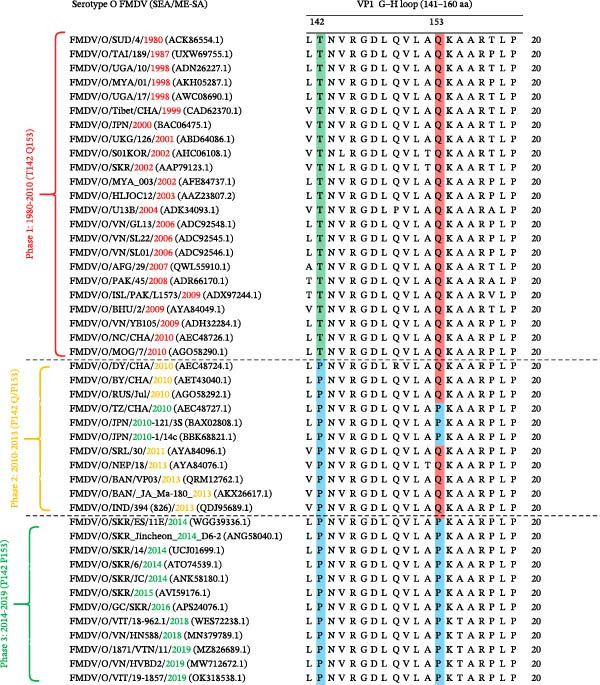
Sequence variations in the G–H loop domain of VP1 protein among representative strains of SEA and ME‐SA topotypes of serotype O FMDV, arranged chronologically by their dates of isolation and identification. This study specifically analyzed amino acid variations at positions 142 and 153 of the VP1 protein in ME‐SA and SEA subtype strains of serotype O FMDV, with a focus on their temporal correlation with isolation dates. Three evolutionary phases: 1980–2010: VP1 position 142 was dominated by threonine (T), and position 153 by glutamine (Q). 2010–2013: All strains exhibited a T → P substitution at position 142, while only a small proportion of strains carried a Q → P substitution at position 153. 2014–2019: The T → P substitution at position 142 became fully fixed, and the Q → P substitution at position 153 was uniformly established across all strains. The key determinants identified at positions 142 and 153 are highlighted in green (T), blue (P), and red (Q) respectively.

In this study, 46 reference sequences of VP1 G–H loop genes from serotype O FMDV strains authenticated by the World Organization for Animal Health (OIE) during the period from 1980 to 2019 were retrieved from the National Center for Biotechnology Information (NCBI, https://www.ncbi.nlm.nih.gov/). The amino acid sequences of the key B‐cell neutralizing epitope VP1 G–H loop (amino acids 141–160) from these 46 serotype O FMDV isolates were sorted by their isolation and identification dates, and comparative analyses were performed using DNAStar MegAlign software (Figure 1). In ME‐SA and SEA lineages of serotype O FMDV, the critical amino acid residues at positions 142 and 153 of the VP1 protein have undergone sequential, stepwise mutations driven by natural selection, which can be divided into three evolutionary phases: 1980–2010: VP1 position 142 was dominated by threonine (T), and position 153 by glutamine (Q). 2010–2013: All strains exhibited a T→P substitution at position 142, while only a small proportion of strains carried a Q→P substitution at position 153. 2014–2019: The T→P substitution at position 142 became fully fixed, and the Q→P substitution at position 153 was uniformly established across all strains. Collectively, these substitutions at the two key residues represent adaptive evolution driven by natural selection. Under the background of intense selective pressure from mandatory vaccination against serotype O FMDV, we hypothesize that these mutations at positions 142 and 153 of the VP1 protein may be associated with viral immune escape. Therefore, subsequent investigations in this study will focus on these two critical amino acid sites of the VP1 protein.

### 2.2. Spatial Localization and Conformational Change Prediction of Key Mutation Sites in VP1 G–H Loop Protein

**Figure 2:**
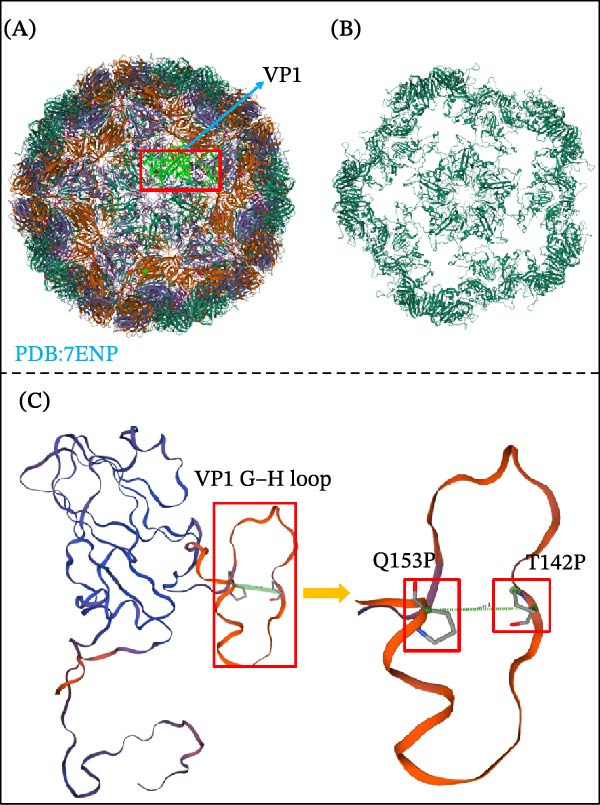
Spatial localization and conformational change prediction of key mutation sites in VP1 G–H loop. (A) Spatial distribution of all structural proteins in FMDV. (B) Spatial distribution of the VP1 protein on FMDV virions. (C) Spatial localization and conformational change prediction of T142P&Q153P mutations in FMDV VP1 structural protein.

To clarify the spatial localization and structural impacts of key mutation sites in the VP1 G–H loop protein, we further predicted the spatial distribution of FMDV structural proteins and conformational changes of mutation sites (Figure 2). Figure 2A illustrates the three‐dimensional spatial arrangement of the full‐length FMDV structural proteins. Figure 2B distinctly shows that the VP1 protein is located on the surface of viral particles, serving as the core structural protein that mediates host immune recognition and regulates antigenic properties. Figure 2C focuses on the critical G–H loop region of the VP1 protein, visually revealing the spatial conformational alterations caused by amino acid mutations at position 142 (Threonine T → Proline P) and position 153 (Glutamine Q → Proline P). The two mutation sites are closely adjacent and both situated in the core neutralizing epitope region of the G–H loop. The introduction of proline alters the folding pattern of peptide chains in this area, triggers the remodeling of local spatial structures, and directly affects the spatial morphology and surface charge distribution of antigenic epitopes. The above structural characteristics confirm that amino acids at positions 142 and 153 of the VP1 protein lie within the key antigenic domain on the viral surface. Their mutations can directly drive conformational variations of antigenic epitopes, providing an important structural basis for the antigenic variation and immune evasion of FMDV.

### 2.3. Construction of Self‐Assembling Nanoparticle Proteins Based on Amino Acid Site Mutants of FMDV 3D Protein T‐Cell Epitopes and VP1 G–H Loop B‐Cell Epitope

The G–H loop domain (amino acid positions 141–160) of the VP1 protein from FMDV is currently recognized as the core linear neutralizing epitope for the development of FMD subunit vaccines [18]. Additionally, the amino acid sequence at positions 56–70 of the FMDV 3D protein, functioning as a highly efficient and specific T‐cell epitope, can be recognized by multiple major histocompatibility complex (MHC) molecules. This T‐cell epitope significantly enhances the immunogenicity of the VP1 protein G–H loop (positions 141–160) epitope in vivo, thereby inducing a potent immune response [19]. Previous studies by our team have confirmed that FMDV nanoparticle vaccine candidates, constructed by fusing the above two key dominant immune epitopes of FMDV with the nanoscaffold protein lumazine synthase (LS) (via separate fusion expression), exhibit an excellent protective immune effect [20].

**Figure 3:**
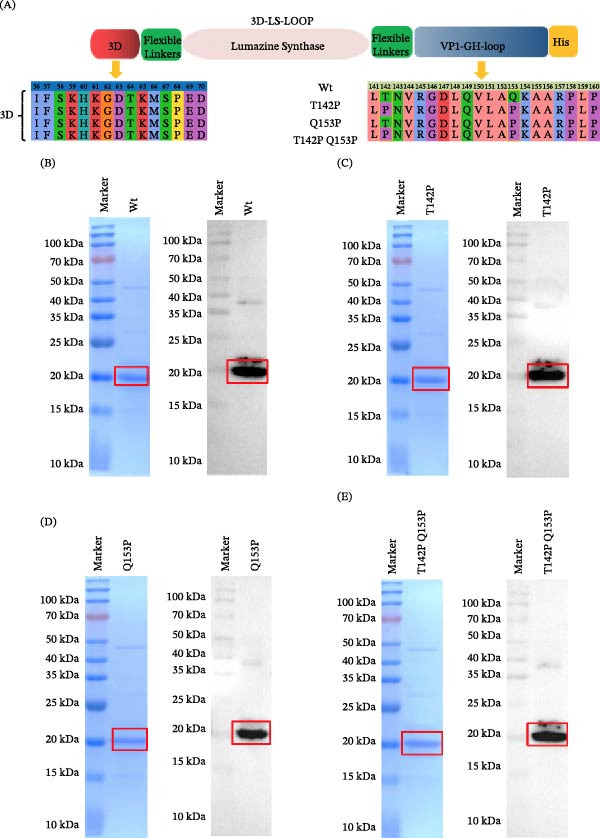
Construction, expression and purification of self‐assembling nanoparticle proteins based on amino acid site mutants of FMDV 3D protein T‐cell epitopes and VP1 G–H loop B‐cell epitope. (A) Schematic diagram of the construction of 3D‐LS‐LOOP mutant self‐assembling nanoparticle protein. (B) SDS‐PAGE and western blot analysis of 3D‐LS‐LOOP (Wt) nanoparticle proteins. (C) SDS‐PAGE and western blot analysis of 3D‐LS‐LOOP (T142P) nanoparticle protein. (D) SDS‐PAGE and western blot analysis of 3D‐LS‐LOOP (Q153P) nanoparticle protein. (E) SDS‐PAGE and western blot analysis of 3D‐LS‐LOOP (T142P&Q153P) nanoparticle protein. SDS‐PAGE analysis was performed by Coomassie brilliant blue staining (left); western blot analysis was performed using a FMDV mAb (pO18‐39) as the primary antibody and horseradish peroxidase‐labeled goat anti‐mouse antibody as the secondary antibody (right). 3D: T‐cell epitope of FMDV 3D protein; LS: lumazine synthase; LOOP: B‐cell epitope of FMDV VP1 G–H loop protein. The 3D‐LS‐LOOP nanoparticle protein can self‐assemble into 60‐mer polymers in vitro.

To systematically investigate the impact of amino acid mutations at positions T142 and Q153 of the VP1 protein on the immunological efficacy of this nanoparticle vaccine, this study first constructed four recombinant plasmids of 3D‐LS‐LOOP mutant nanoparticles using site‐directed mutagenesis technology (Figure 3A). The detailed experimental procedures are as follows: the genes encoding the aforementioned mutant nanoparticle proteins were directionally cloned into the pET‐28a prokaryotic expression vector, which was then transformed into competent *Escherichia coli* (*E. coli*) BL21 (DE3) cells. An isopropyl *β*‐D‐1‐thiogalactopyranoside (IPTG)‐inducible expression system was employed to achieve the induced expression of the target proteins. Results from in vitro assembly verification showed that the expressed mutant nanoparticle proteins could self‐assemble into 60‐mer supramolecular structures, consistent with the structural characteristics of nanoparticle vaccines.

For the purification of target proteins, Ni‐NTA affinity chromatography was utilized. The target proteins were specifically captured and purified from the supernatant of lysed BL21 (DE3) cells under low‐temperature conditions (4°C) to minimize protein denaturation and maintain their biological activity. After purification, the FMDV VP1 protein‐specific monoclonal antibody (pO18‐39) was used as the primary antibody. Protein purity was determined by sodium dodecyl sulfate‐polyacrylamide gel electrophoresis (SDS‐PAGE), and the specific expression of the target proteins was verified by western blotting analysis. The results demonstrated that all four mutant nanoparticle proteins were efficiently expressed; with reference to protein molecular weight standards, their molecular weights were ~23 kDa (Figure 3B–E). In subsequent experiments, neutralizing antibody cross‐protection tests will be conducted with four rescued FMDV mutant strains to further clarify the regulatory mechanism of amino acid mutations at positions T142 and Q153 on the immunological efficacy of this.

### 2.4. Generation and Comprehensive Characterization of Serotype O FMDV VP1 Mutants

To further substantiate the importance of VP1 residues T142 and Q153, the present study established infectious clone strains harboring mutations at these sites in the FMDV O/MYA/01/1998 (AKH05287.1) strain background, which are viable in both BSR/T7 and BHK‐21 cell cultures. This was accomplished via unidirectional assembly of the full‐length genomic cDNA of the FMDV O/MYA/01/1998 strain, using the pBlue script Ⅱ plasmid containing the complete P1 gene of the FMDV O/MYA/01/1998 strain as the template.

**Figure 4:**
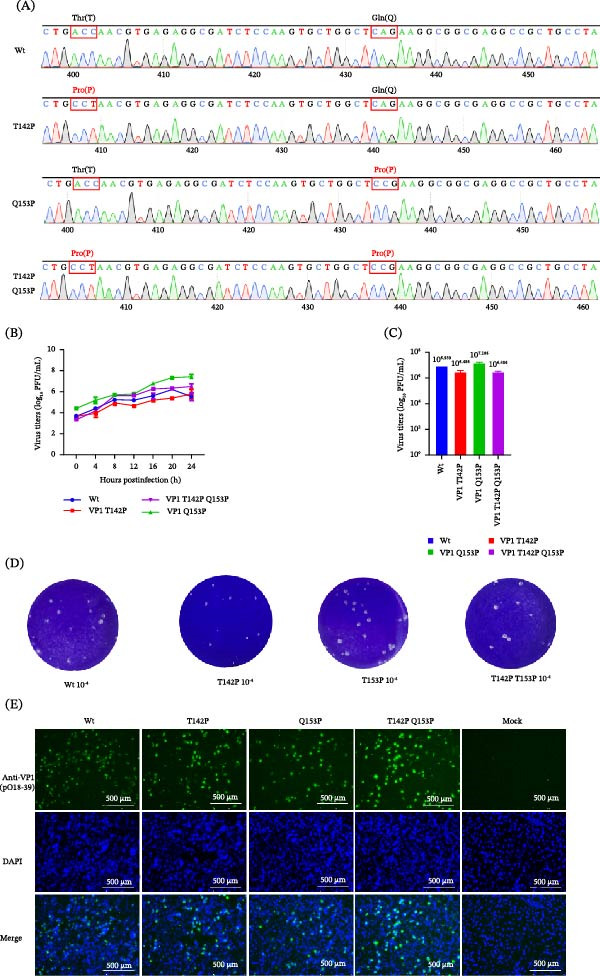
Analysis of virological characteristics of FMDV O/MYA/01/1998 (Wt) strain following key amino acid substitutions in the VP1 G–H loop (T142P, Q153P, and T142P&Q153P). (A) Analysis of Sanger sequencing chromatograms for the VP1 G–H loop of Wt and rescued mutant viruses. (B) One‐step growth kinetics analysis of Wt and rescued mutant strains BHK‐21 monolayer cells were seeded in 24‐well plates and infected with Wt and mutant strains at a multiplicity of infection (MOI) of 1.0, respectively. Samples were collected at 0, 4, 8, 12, 16, 20, and 24 h postinfection, and the average log_10_ titers at each time point were determined by the TCID_50_ assay. (C) Plaque assay to determine the viral titers (PFU/mL) of Wt and mutant strains. (D) Plaque phenotypes of Wt and mutant strains in BHK‐21 cells at 48 h postinfection (h.p.i.)., with their sizes correlated to cytopathic effect (CPE) patterns. (E) Indirect Immunofluorescence Assay (IFA) identification and analysis of Wt and rescued mutant strains. BHK‐21 cells were infected with viruses (including Wt and all mutant strains) at an MOI of 1.0. At 10 h postinfection, the cells were assayed using a porcine anti‐VP1 monoclonal antibody (pO18‐39) as the primary antibody and an FITC‐conjugated porcine secondary antibody for the detection of FMDV VP1 protein. Scale bars are shown in each image.

Nucleotide mutations were introduced through site‐directed mutagenesis to generate full‐length cDNA constructs with either single (T142P or Q153P) or double (T142P/Q153P) amino acid substitutions (Figure S1). The correctness of all mutant constructs was verified by nucleotide sequencing. Mutant plasmids were then linearized with Not I and transfected into BSR/T7 cells using Lipofectamine 3000 according to the manufacturer’s instructions (Figure S2). Transfected cells were monitored daily for the presence of cytopathic effects (CPEs). At 72 h posttransfection, culture supernatants were collected and serially passaged in BHK‐21 cells. The rescued viruses were designated as Wt (FMDV O/MYA/01/1998), T142P, Q153P, and T142P/Q153P, respectively. Following plaque purification (Figure 4D), monoclonal populations of each virus were propagated in BHK‐21 cells, and VP1 gene sequencing confirmed that the genomic sequences were consistent (Figure 4A). Subsequent analyses of viral growth kinetics and titers (Figure 4B, C) demonstrated that these mutations had minimal impact on viral titers and growth trends. Furthermore, indirect immunofluorescence staining confirmed the presence of the VP1 protein in BHK‐21 cells infected with Wt, T142P, Q153P, or T142P/Q153P (Figure 4E). Collectively, these results validate the successful rescue of the Wt and mutant FMDV strains.

### 2.5. Study on the Differences in Humoral Immune Efficacy Induced by FMDV Mutant Nanoparticle Proteins in C57BL/6 Mice

**Figure 5:**
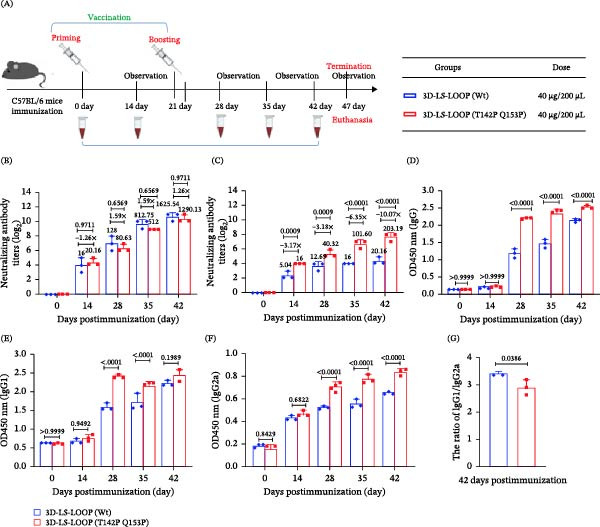
Evaluation of serum neutralizing antibody‐mediated cross‐protection against prevalent FMDV O/MYA/01/1998 (AKH05287.1) and FMDV O/TZ/CHA/2010 (AEC48727.1) strain strain of serotype O FMDV after immunization of C57BL/6 mice with 3D‐LS‐LOOP nanoparticle vaccines based on Wt and double‐mutant strain amino acid sites in the FMDV VP1 G–H loop. (A) Schematic diagram of immunization protocol and dosage for C57BL/6 mice. C57BL/6 mice were prime/boost‐vaccinated with vaccines at 0 day and 21 days. Blood was collected at 0, 14, 28, 35, and 42 dpi. All mice were euthanized at 47 dpi. (B) Determination of nAb titers against the Wt strain (FMDV O/MYA/01/1998) in the serum of each immunized mouse at specified time intervals. (C) Determination of nAb titers against the double‐mutant strain (FMDV O/TZ/CHA/2010) in the serum of each immunized mouse at specified time intervals. (D–F) Detection of FMDV‐specific IgG, IgG1 and IgG2a antibodies levels at different time points by Indirect ELISA. (G) The ratio of FMDV‐specific IgG1 and IgG2a antibodies titers at 42 dpi by Indirect ELISA. Data in subparts B–G are presented as mean ± SD. Statistical differences were analyzed via one‐way ANOVA followed by Tukey’s multiple comparisons test. All experiments were performed in triplicate (*n* = 5 mice for each group), and exact *p* values are displayed.

This study aimed to analyze the effects of mutations at the T142 and Q153 sites of the FMDV VP1 protein on humoral immunity and neutralizing antibody responses induced by its G–H loop (B‐cell neutralizing epitope) in mice. Based on the previously established 3D‐LS‐LOOP nanoparticle vaccine scaffold, this research retained the immunodominant T‐cell epitope 3D and the nanoparticle scaffold LS and only performed site‐directed mutagenesis on the target sites of the G–H loop [20]. Two immunogens, 3D‐LS‐LOOP (Wt) and double‐mutant 3D‐LS‐LOOP (T142P&Q153P), were constructed. First, the levels of humoral immunity induced by the two immunogens were detected (Figure 5). The results showed that both proteins could induce high‐titer neutralizing antibodies against the FMDV O/MYA/01/1998 strain in mice, with a peak titer of 1:2^10.66^, and there was no statistically significant difference between groups (*p* = 0.9711). The antibody titers kept rising throughout the immunization period and reached the peak on Day 42 postimmunization. For the FMDV O/TZ/CHA/2010 strain, the overall neutralizing antibody levels of both groups were relatively low. On Day 42 after immunization, the antibody titer of the 3D‐LS‐LOOP (T142P&Q153P) group was 1:2^7.67^, ~10 times higher than that of the 3D‐LS‐LOOP (Wt) group (1:2^4.33^), presenting an extremely significant difference between groups (*p* < 0.0001).

Further detection of virus‐specific IgG and its subtypes IgG1 and IgG2a was conducted (Figure 5D–F). No obvious difference was observed in antibody levels between the two groups within 14 days after the primary immunization (*p* > 0.05). After the booster immunization on Day 21, the levels of all types of antibodies in the 3D‐LS‐LOOP (T142P&Q153P) group were markedly higher than those in 3D‐LS‐LOOP (Wt) group (*p* < 0.0001), indicating a superior humoral immune effect. Subtype analysis also confirmed that both immunogens could induce a Th1‐biased immune response in mice (Figure 5G).

**Figure 6:**
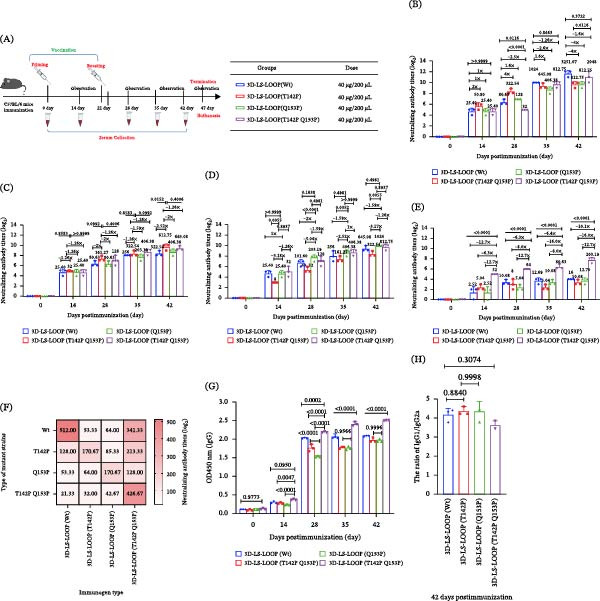
Evaluation of cross‐protection mediated by serum neutralizing antibodies against foot‐and‐mouth disease virus VP1 G–H loop isogenic mutant rescued strains after immunization of C57BL/6 mice with 3D‐LS‐LOOP nanoparticle vaccines based on VP1 G–H loop amino acid site mutations (T142P&Q153P). (A) Schematic diagram of immunization protocol and dosage for C57BL/6 mice. C57BL/6 mice were prime/boost‐vaccinated with vaccines at 0 day and 21 days. Blood was collected at 0, 14, 28, 35, and 42 dpi. All mice were euthanized at 47 dpi. (B) Determination of neutralizing antibody (nAb) titers against the FMDV (Wt) strain in the serum of each immunized mouse at specified time intervals. (C) Determination of nAb titers against the FMDV single mutant (T142P) strain in the serum of immunized mice at specified time intervals. (D) Determination of nAb titers against the FMDV single mutant (Q153P) strain in the serum of each immunized mouse at specified time intervals. (E) Determination of nAb titers against the FMDV double mutant (T142P&Q153P) strain in the serum of each immunized mouse at specified time intervals. (F) The cross‐protective efficacy of the 3D‐LS‐LOOP nanoparticle vaccines based on VP1 G–H loop amino acid site mutations (T142P& Q153P) in inducing mice serum NAbs titers against prevalent FMDV O/MYA/01/1998 (AKH05287.1) strain and FMDV rescue strains. (G) Detection of FMDV‐specific IgG antibodies levels at different time points by Indirect ELISA. (H) The ratio of FMDV‐specific IgG1 and IgG2a antibodies titers at 42 dpi by Indirect ELISA. Data in subparts B–H are presented as mean ± SD. Statistical differences were analyzed via one‐way ANOVA followed by Tukey’s multiple comparisons test. All experiments were performed in triplicate (*n* = 5 mice for each group), and exact *p* values are displayed.

To explore the effects of single amino acid mutations, two single‐mutant nanoparticle proteins, T142P and Q153P, were further constructed in this study. Four candidate vaccines were prepared by combining the four types of nanoparticles with ISA 201 VG adjuvant, and their efficacy was evaluated in C57BL/6 mice following the original immunization protocol (Figure 6A).

The immunization results (Figure 6B–D) demonstrated that all four vaccines could elicit high levels of neutralizing antibodies against the Wt strain and the two single‐mutant strains. The variation trend of antibodies was consistent with previous results, with the peak appearing on Day 42 postimmunization. However, when targeting the T142P&Q153P double‐mutant strain, the overall neutralizing antibody titers decreased significantly. There was no statistical difference among the 3D‐LS‐LOOP (Wt) vaccine group and the two single‐mutant vaccine groups (*p* > 0.05), while the antibody titer of 3D‐LS‐LOOP (T142P&Q153P) vaccine group was 10–16 times higher than those of the other three groups, showing an extremely significant difference (*p* < 0.0001, Figure 6E). The detection results of IgG and its subtypes further verified that the 3D‐LS‐LOOP (T142P&Q153P) vaccine exerted a remarkably better humoral immune effect than the other three vaccines, and all four vaccines were capable of inducing a Th1‐biased immune response (Figure 6G, H).

In conclusion, single‐site mutations at T142 or Q153 of the VP1 protein barely affected the vaccine‐induced humoral immune response, whereas the combined mutation of the two sites could markedly enhance the humoral immune capacity of the vaccine. Nevertheless, the T142P/Q153P double mutation would lead to viral immune escape and reduce the protective efficacy of the vaccine against this mutant strain.

### 2.6. Differential Analysis of Cross‐Protective Efficacy of Neutralizing Antibodies Induced by FMDV Nanoparticle Proteins and Multiple Commercial Inactivated Vaccines in Pigs

To further validate the differences in NAb titers and cross‐protective efficacy induced in pigs between our developed nanoparticle candidate vaccines and commercial inactivated vaccines—with specific focus on the four FMDV strains harboring mutations at the two key VP1 residues (T142 and Q153)—we designed a head‐to‐head comparative study.

Three widely used commercial inactivated FMD vaccines for pigs in China were selected as reference controls: FMDV Serotypes O/A Commercial Inactivated Vaccine A: Porcine FMD Bivalent Inactivated Vaccine (Serotype O: O/MYA98/BY/2010 strain + O/PanAsia/TZ/2011 strain; Serotype A: Re‐A/WH/09 strain); FMDV Serotypes O/A Commercial Inactivated Vaccine B: FMD Bivalent Inactivated Vaccine (Serotype O: O/MYA98/BY/2010 strain; Serotype A: Re‐A/WH/09 strain), containing inactivated FMD virus of O/MYA98/BY/2010 (Serotype O) and Re‐A/WH/09 (Serotype A). FMDV Serotypes O Commercial Inactivated Vaccine C: Porcine FMD Monovalent Inactivated Vaccine (Serotype O: O/Mya98/XJ/2010 strain+O/GX/09‐7 strain), with main components being inactivated FMD virus of O/Mya98/XJ/2010 and O/GX/09‐7 (both Serotype O).

**Figure 7:**
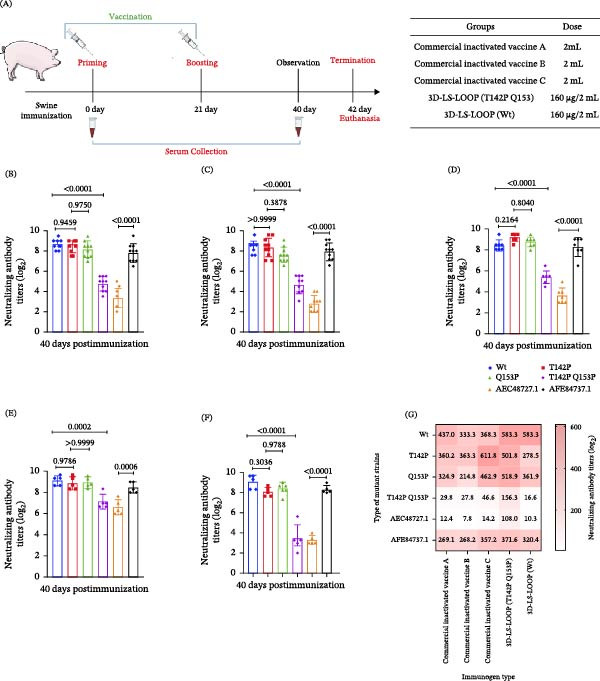
Evaluation of cross‐protective efficacy of neutralizing antibodies against FMDV rescue strains with different amino acid site mutations in the VP1 protein G–H loop with multiple FMDV vaccines. (A) Schematic diagram of immunization protocol and dosage for swine. The primary/booster immunization were conducted respectively on Day 0 and Day 21. Blood samples were only collected at 0 and 40 dpi. All swine were euthanized at 42 dpi. (B–F) Evaluation of cross‐protective efficacy of neutralizing antibodies against FMDV rescue strains with different amino acid site mutations in the VP1 protein G–H loop at 40 dpi of swine with multiple FMDV commercial inactivated vaccines [(B‐D) FMDV serotypes O/A commercial inactivated vaccine A‐C] and FMDV nanoparticle vaccines [(E) 3D‐LS‐LOOP (T142P&Q153P) and (F) 3D‐LS‐LOOP (Wt)]. (G) The cross‐protective efficacy of the multiple FMDV vaccines in inducing swine serum NAbs titers against prevalent FMDV Wt strain (GenBank: AEC48727.1 and AFE84737.1) and FMDV rescue strains. Data in subparts B–F are presented as mean ± SD. Statistical differences were analyzed via one‐way ANOVA followed by Tukey’s multiple comparisons test. All experiments were performed in triplicate (*n* = 5/10 pigs for each group), and exact *p* values are displayed.

These commercial inactivated vaccines were compared with our two FMDV nanoparticle candidates: 3D‐LS‐LOOP (Wt) and 3D‐LS‐LOOP (T142P&Q153P). Pigs were immunized with each vaccine on Day 0 (prime) and Day 21 (booster), and NAb titers against the four rescued FMDV strains (Wt, T142P, Q153P, and T142P/Q153P) were determined at 40 days postinfection (dpi) (Figure 7A). As shown in Figure 7B–F, three commercially available inactivated vaccines and two candidate nanoparticle vaccines all induced high levels of neutralizing antibodies against the Wt rescued strain, T142P single mutant rescued strain, Q153P single mutant rescued strain, and the wild‐type strain (GenBank: AFE84737.1) in experimental pigs, with antibody titers ranging from 1:2^8.06^ to 1:2^9.26^ No significant differences were observed in the neutralizing antibody titers elicited by the five vaccine groups against the aforementioned strains (*p* > 0.05). When detecting neutralizing antibodies against the T142P/Q153P double mutant rescued strain and the double mutant wild strain (GenBank: AEC48727.1), significant differences were found among all groups. Except for the 3D‐LS‐LOOP (T142P&Q153P) candidate nanoparticle vaccine, all other vaccines induced low neutralizing antibody titers, ranging only from 1:2^2.96^ to 1:2^5.54^. In contrast, the 3D‐LS‐LOOP (T142P&Q153P) vaccine induced high levels of neutralizing antibodies, with titers of 1:2^7.29^ and 1:2^6.75^ against the two double mutant strains, respectively, and the differences between groups were extremely significant (*p* < 0.0001).

As illustrated in Figure 7G, the 3D‐LS‐LOOP (T142P&Q153P) vaccine exhibited better neutralizing efficacy against the Wt strain and T142P and Q153P single mutant strains than the three commercially available inactivated vaccines and the 3D‐LS‐LOOP (Wt) candidate vaccine. It also exerted favorable neutralizing and protective effects against the T142P/Q153P double mutant strain. In conclusion, the combined mutations of T142P and Q153P in the VP1 protein are key sites for immune escape of topotype O FMDV prevalent in the SEA and ME‐SA. Immunogens carrying these combined mutations can provide efficient cross‐neutralization and protection against wild strains and double mutant strains, which verifies that the introduction of VP1 T142P/Q153P combined mutations can effectively broaden the antigenic spectrum of vaccines. In conclusion, mutations at the key sites T142 and Q153 of VP1 can not only regulate vaccine‐mediated humoral immune responses but also induce the T142P/Q153P mutant strain to develop an immune escape phenotype. Moreover, variations at these sites can reduce the protective efficacy of commercially available conventional inactivated vaccines, thereby facilitating the progression of viral immune escape. The findings of this study can provide an important reference for the subsequent optimization and formulation of FMDV prevention and control strategies.

## 3. Discussion

FMDV can evade immune responses through adaptive evolution under vaccine‐induced selective pressure, leading to recurrent infections in vaccinated hosts and posing a persistent threat to global livestock security [21]. The VP1 protein of FMDV exhibits high accessibility to the host immune system and contains a receptor‐binding site (the RGD motif) within its G–H loop; this site can undergo continuous changes under immune pressure, thereby contributing to the emergence of new epidemic strains [22]. In this study, we analyzed 46 serotype O FMDV strains of SEA and ME‐SA topotypes retrieved from the NCBI database (1980–2019), with a focus on the VP1 G–H loop (amino acids 141–160)—a key neutralizing epitope of the virus. Sequence analysis revealed the time‐dependent accumulation of site‐specific mutations at VP1 residues 142 (threonine → proline, T → P) and 153 (glutamine → proline, Q → P). To verify the functional impacts of these mutations on viral strains and address the core question of “whether mutations affect vaccine protective efficacy,” we integrated the aforementioned mutations into the well‐validated and highly efficient 3D‐LS‐LOOP nanoparticle candidate vaccine. The mutant proteins were then efficiently expressed and purified using an *E coli* prokaryotic expression system.

We first evaluated the humoral immunogenicity of two FMD nanoparticle proteins‐3D‐LS‐LOOP (Wt) and 3D‐LS‐LOOP (T142P&Q153P), using a C57BL/6 mouse model. Results demonstrated that both candidate vaccines effectively protected against the Wt strain (FMDV O/MYA/01/1998); however, their protective efficacy was significantly reduced when challenged with the double‐mutant strain (FMDV O/TZ/CHA/2010). To further dissect the impact of single mutations at the two key VP1 residues (T142 and Q153) on the humoral immune response in mice, we constructed two additional single‐mutant nanoparticle candidates: 3D‐LS‐LOOP (T142P) and 3D‐LS‐LOOP (Q153P). Additionally, to assess the effect of these site‐specific mutations on viral characteristics while ensuring experimental consistency, we generated infectious FMDV clones harboring these mutations in the genetic background of the FMDV O/MYA/01/1998 strain. Nucleotide mutations were introduced via site‐directed mutagenesis to construct full‐length cDNA clones with either single (T142P or Q153P) or double (T142P/Q153P) amino acid substitutions, from which four FMDV strains (Wt, T142P, Q153P, and T142P/Q153P) were successfully rescued.

Subsequently, we evaluated the cross‐protective efficacy of the four nanoparticle candidates by measuring NAb titers induced in mice against the four rescued strains. All four candidates induced high NAb titers against the rescued Wt and single‐mutant strains, but NAb titers against the T142P/Q153P double‐mutant strain were reduced across all groups. In contrast, the 3D‐LS‐LOOP (T142P/Q153P) candidate exhibited a unique advantage: its induced NAb titers against the double‐mutant strain was significantly more than 10‐fold higher than that of the other nanoparticle candidates (*p* < 0.0001). More importantly, a similar trend was observed in pigs immunized with three commercial inactivated FMD vaccines: all three commercial vaccines induced only low NAb titers against the double‐mutant strain, whereas the double‐mutant nanoparticle candidate maintained high‐titer NAb production. This cross‐species consistency—from mice to pigs—indicates that the antigenic alteration mediated by the T142P/Q153P mutation is host‐independent, and the strategy of “incorporating immune escape mutations into vaccine antigens” possesses broad applicability. Collectively, these findings further confirm that the combined mutation of VP1 residues 142 and 153 is likely to alter FMDV antigenicity, whereas single‐residue mutations exert minimal effects on viral antigenicity.

While the complete protective mechanism of FMD vaccines has not yet been fully elucidated, the core role of NAbs in preventing FMDV infection following vaccination has been well‐documented [23]. To clarify the mechanisms underlying antibody‐mediated neutralization of infection and viral evasion of immune recognition, it is not only necessary to define the overall structure of the virus but also to conduct in‐depth analysis of virus–antibody interaction details at the amino acid level [24, 25]. The amino acids at positions 142 and 153 of the VP1 protein have special functional significance as both are located in the core region of the major neutralizing epitope within the VP1 G–H loop (Figure 3). Mutations at these positions can directly alter the spatial conformation and charge distribution of the epitope: compared with Wt threonine (T) and glutamine (Q), proline (P) introduces a rigid kink into the peptide chain, thereby disrupting the inherent flexible structure of the epitope. Meanwhile, the loss of polar side chains—such as the hydroxyl group of threonine and the amide group of glutamine—leads to the redistribution of surface charge on the epitope, which significantly impairs the electrostatic interactions with the complementarity‐determining regions (CDRs) of neutralizing antibodies. These two effects collectively reduce the binding affinity between antibodies and the virus to a significant extent, ultimately resulting in decreased neutralizing activity [26, 27]. Notably, when combined mutations occur at both positions, the local spatial structural changes of the antigenic epitope become more pronounced, further hindering the tight binding between antibodies and the epitope and enabling the virus to more easily evade recognition and attack by the host immune system [28, 29].

Current FMD subunit vaccines are mostly designed based on the antigenic epitopes of the Wt strain. When combined mutations occur at amino acid positions 142 and 153 of the viral structural protein VP1, the antibodies induced by the vaccine fail to properly match the mutated epitopes, resulting in reduced binding capacity to the virus. This leads to neutralization failure and a significant decline in the vaccine’s protective efficacy against the mutant strain and ultimately enables the virus to survive, replicate in the host, and trigger immune escape [30]. Therefore, an in‐depth investigation of the impact of combined mutations at these two positions on FMDV antigenicity and immune escape ability is of great significance for elucidating the virus’s pathogenic and immune escape mechanisms.

This study verified that combined mutations at VP1 positions 142/153 exert a far greater impact on viral antigenicity than single mutations; the synergistic effect of these two mutations drives complex changes in the VP1 protein structure and epitopes that cannot be achieved by single‐site mutations. Unlike previous studies, which mostly explored the effects of mutations in scattered regions of VP1, this study focuses on the specific “immune escape hotspot” at VP1 positions 142/153, clarifying its accumulative characteristics during evolution and its key impact on vaccine efficacy. This not only provides a new perspective for understanding the molecular mechanism of VP1 protein antigenicity but also fills the research gap regarding “how FMDV achieves immune escape through multisite synergistic mutations under long‐term vaccine pressure.” For current commercial inactivated FMD vaccines based on Wt strains, the results of this study explain the reason for their reduced protective efficacy against circulating mutant strains and provide a clear direction for the design of next‐generation FMDV vaccines. In summary, combined mutations at VP1 positions 142/153 of FMDV are a key event enabling the virus to achieve immune escape under vaccine pressure, as they synergistically alter the structure of antigenic epitopes and lead to decreased protective efficacy of traditional vaccines [30]. In contrast, the nanoparticle vaccine designed based on these mutations can effectively address this issue. These findings not only deepen our understanding of FMDV evolution mechanisms but also confirm that incorporating immune escape hotspot mutations into vaccine antigens can expand the antibody reactive spectrum, providing experimental evidence for the development of next‐generation broad‐spectrum FMDV vaccines and holding great significance for global FMD prevention and control in the livestock industry.

## 4. Material and Methods

### 4.1. Ethics Statement

All animal experiments were strictly conducted in accordance with the animal ethics guidelines of the Animal Experiment Inspection Bureau and the Ethics Review Committee and approved by the Animal Ethics Committee of Huazhong Agricultural University and have obtained animal welfare approval from the College of Veterinary Medicine, Huazhong Agricultural University, Hubei Province, China (Ethics Number: HZAUMO‐2022‐0214 for mouse experiments; YFXJ‐2023‐002 for pig experiments). All operations involving live FMDV were performed in the Biosafety Level 3 Laboratory of the Lanzhou Veterinary Research Institute, Chinese Academy of Agricultural Sciences.

### 4.2. Virus and Animals

The FMDV O/MYA/01/1998 (GenBank Accession Number: AKH05287.1, Wt) strain, FMDV O/TZ/CHA/2010 (GenBank Accession Number: AEC48727.1) strain, and FMDV O/MYA/003/2002 (GenBank Accession Number: AFE84737.1) were obtained from the National FMD Reference Laboratory, Lanzhou Veterinary Research Institute, Chinese Academy of Agricultural Sciences. Specific pathogen‐free (SPF) C57BL/6 mice were acquired from the College of Veterinary Medicine, Huazhong Agricultural University, Hubei, China. FMDV‐negative pigs were provided by Guangxi Yangxiang Group Co., Ltd., China.

### 4.3. Sequence Acquisition and Analysis of VP1 G–H Loop From Serotype O FMDV

We retrieved 46 VP1 G–H loop gene sequences of serotype O FMDV strains belonging to SEA or ME‐SA topotypes (1980–2019) from the NCBI database [31]. Amino acid sequence analysis and alignment of the VP1 G–H loop were performed using DNAStar MegAlign software [32].

### 4.4. Rescue of FMDV VP1 Site‐Directed Mutants Using Reverse Genetics

Full‐length cDNAs were constructed using the pBlue‐script Ⅱ plasmid, which harbors the complete P1 gene of the FMDV O/MYA/01/1998 strain, as the template. Nucleotide mutations were introduced via site‐directed mutagenesis to generate full‐length cDNAs containing single‐ or double‐amino acid substitutions [33]. The correctness of the mutant constructs was verified by nucleotide sequencing. FMDV site‐directed mutants were rescued following established protocols [34], with detailed procedures as follows: mutant plasmids were linearized by Not I digestion and transfected into BSR/T7 cells using Lipofectamine 3000 according to the manufacturer’s instructions. Transfected cells were monitored daily for the presence of the CPE. At 72 h posttransfection, culture supernatants were collected, passaged in BHK‐21 cells, and viral titers were determined.

### 4.5. Indirect Immunofluorescence Assay (IFA)

The rescued virus strains were detected using IFA. BHK‐21 cells (2 × 10^6^ cells/mL) were seeded into 6‐well plates and infected with Wt and mutant strains at a multiplicity of infection (MOI) of 1. At 12 h postinfection (h.p.i.), cells were washed 3 times with PBST, fixed with 4% paraformaldehyde for 30 min at room temperature, and permeabilized with Triton X‐100 for 20 min at 20°C. After blocking with 5% bovine serum albumin (BSA) for 2 h at room temperature, cells were incubated with pO18‐39 monoclonal antibody (1:1000) at 37°C for 1.5 h. Following 3 washes with PBST, cells were incubated with FITC‐conjugated goat anti‐pig secondary antibody at 37°C for 1 h. After thorough washing, nuclei were stained with DAPI (Beyotime Biotechnology, Shanghai, China), and cells were observed and imaged under a fluorescence microscope (Ti‐U‐Nikon, Tokyo, Japan) [35].

### 4.6. Viral Growth Curve Assay

BHK‐21 cells seeded in 24‐well plates were washed twice with DMEM and then infected with FMDV at a MOI of 0.1. After viral adsorption at 4°C for 1 h, the medium was removed, and cells were washed once more with DMEM before incubation in DMEM supplemented with 2% fetal bovine serum (FBS). At 0, 4, 8, 12, 16, 20, and 24 h.p.i., cultures were harvested by three cycles of freeze‐thawing. Viral titers were determined using the 50% tissue culture infective dose (TCID_50_) assay [36].

### 4.7. TCID_50_ Assay

BHK‐21 cells in 96‐well plates were washed once with DMEM, followed by the addition of 100 μL serially diluted virus (in DMEM) to each well (8 replicate wells per dilution). After 1 h, 100 μL DMEM supplemented with 2% FBS was added to each well. CPEs were observed and recorded under a microscope at 3–5 dpi, and TCID_50_ titers were calculated using the Reed‐Muench method [37].

### 4.8. Plasmid Construction of Mutant Nanoparticle Proteins

The gene fragment encoding LS was synthesized. Specific primers containing Nco I and Xho I restriction enzyme sites were designed. Using LS as the template, PCR was performed to amplify the nucleotide sequences corresponding to the T‐cell epitope 3D (residues 56–70) and the B‐cell epitope VP1 G–H loop (residues 141–160) in segments. These sequences were linked to the N‐terminus and C‐terminus of LS via a GS‐linker, respectively. After enzymatic digestion, the 3D‐LS‐LOOP gene fragment was cloned into the Nco I/Xho I restriction sites of the pET‐28a plasmid. The recombinant plasmid was transformed into *Escherichia coli* (*E. coli*) DH5α (Stratagene, USA) to obtain pET‐28a‐3D‐LS‐LOOP (Wt). Subsequent site‐directed mutagenesis was performed using a site‐mutation kit, followed by transformation into *E. coli* DH5α to generate pET‐28a‐3D‐LS‐LOOP (T142P), pET‐28a‐3D‐LS‐LOOP (Q153P), and pET‐28a‐3D‐LS‐LOOP (T142P&Q153P).

### 4.9. Expression and Purification of Nanoparticle Proteins

Recombinant plasmids were transformed into *E. coli* BL21 cells (Stratagene, USA). Bacterial cultures were grown in LB medium supplemented with 50 μg/mL kanamycin, with shaking at 180 rpm at 37°C. When the optical density at 600 nm (OD_600_) reached 0.6–0.8, isopropyl‐*β*‐D‐thiogalactopyranoside (IPTG) was added to a final concentration of 0.4 mM, and cultures were incubated for an additional 6 h under the same conditions (37°C, 180 rpm). Bacterial pellets were resuspended in Tris‐HCl buffer (pH 8.0), followed by low‐temperature ultrasonic disruption. The supernatant was centrifuged at 12,000 rpm for 20 min at 4°C and then filtered through a 0.22 μm membrane. Purification was performed using Ni Sepharose 6 Fast Flow resin (GE Healthcare, Shanghai, China), with protein elution carried out using a protein purification system (Bio‐Rad, USA). Further analysis of nanoparticle proteins was conducted via size‐exclusion chromatography (SEC) using a HiLoad 16/600 Superdex 75 prep grade column (GE Healthcare, Shanghai, China). Protein purity was determined by 12% SDS‐PAGE.

### 4.10. SDS‐PAGE and Western Blot Analysis

The molecular weight of nanoparticle proteins was confirmed by SDS‐PAGE and western blot, as previously described. Protein samples were collected, resuspended in 5× loading buffer, boiled at 95°C for 10 min, and then placed on ice for 2 min. For SDS‐PAGE, gels were stained with Coomassie brilliant blue (Ebos Biological, China) and destained for 1 h. For western blot, the pO18‐39 (VP1) monoclonal antibody (1:5000 dilution) was used as the primary antibody, and horseradish peroxidase (HRP)‐conjugated goat anti‐pig IgG (AB Clone, China) served as the secondary antibody to detect specific protein bands. Protein bands were visualized using the Bio‐Rad Chemi Doc XRS + imaging system with an enhanced chemiluminescence (ECL) kit (BeyoECL Plus, Biyuntian, China) and analyzed with Image Lab 4.0.1 software (Bio‐Rad, USA) [38].

### 4.11. Virus Neutralization Assay

Viral neutralizing antibody titers against FMDV were determined using the MN test, as described previously [39]. Briefly, inactivated sera were heated at 56°C for 30 min and then serially diluted 2‐fold in 96‐well cell culture plates in a total volume of 50 μL. Each well was then supplemented with 50 μL of medium containing 100 50% tissue culture infective doses (TCID_50_) of FMDV. After incubation at 37°C for 1 h, ~5 × 10^4^ BHK‐21 cells suspended in 100 μL of the medium were added to each well. Plates were subsequently incubated at 37°C with 5% CO_2_ for 72 h, after which CPEs were observed. The endpoint titer was calculated as the reciprocal of the last serum dilution that neutralized 100 TCID_50_ of FMDV in 50% of the wells, expressed as the log_2_ reciprocal of the highest serum dilution inhibiting 50% of FMDV replication.

### 4.12. Specific IgG Antibody Analysis

An indirect enzyme‐linked immunosorbent assay (ELISA) was used to detect FMDV‐specific IgG, IgG1, and IgG2a antibodies in mouse. Purified FMDV‐VP1 (0.25 μg/well) was diluted in 0.05 M carbonate‐bicarbonate buffer (pH 9.6) and coated onto 96‐well microtiter plates. After blocking with 1% BSA at 37°C for 1 h, plates were washed 3 times with phosphate‐buffered saline containing 0.1% Tween‐20 (PBS‐T). Next, 100 μL of serum samples (diluted 1:3600) were added to each well and incubated overnight at 4°C. Subsequently, HRP‐conjugated goat anti‐mouse IgG (1:5000), IgG1 (1:10000), or IgG2a (1:10000) was added, followed by incubation at 37°C for 1 h. After 3 washes with PBS‐T, 100 μL of tetramethylbenzidine (TMB; Solarbio Science & Technology, Beijing, China) was added and incubated at 37°C for 15 min. The reaction was terminated by adding a stop solution (Solarbio Science & Technology, Beijing, China), and absorbance was measured at 450 nm using a multimode microplate reader (Tecan Spark 10 M) [38].

### 4.13. Mouse Immunization Experiment Plan

Six‐week‐old female SPF‐grade C57BL/6 mice were used in this experiment, and all experimental mice were purchased from the Laboratory Animal Research Center of Huazhong Agricultural University in Wuhan. The antigen was diluted to 400 μg/mL with phosphate‐buffered saline and then mixed and emulsified with Montanide ISA 201 VG adjuvant at a volume ratio of 1:1 to prepare the experimental vaccine with a final concentration of 200 μg/mL.

In the primary immunization experiment, the mice were divided into two groups: the 3D‐LS‐LOOP (Wt) group and the 3D‐LS‐LOOP (T142P&Q153P) double mutant group, with 5 mice in each group. The detailed immunization protocol is shown in Table S1. In the subsequent experiments, the mice were randomly assigned to four groups, namely, the 3D‐LS‐LOOP (Wt) group, the 3D‐LS‐LOOP (T142P) single mutant group, the 3D‐LS‐LOOP (Q153P) single mutant group, and the 3D‐LS‐LOOP (T142P&Q153P) double mutant group, each containing 5 mice. The corresponding immunization protocols are presented in Table S2.

All mice were immunized via an intramuscular injection at a dose of 200 μL per mouse, containing 40 μg of antigen. The phosphate‐buffered saline control group. The primary immunization was performed on Day 1 of the experiment, and a booster immunization with the same dose was administered 3 weeks later. Serum samples were collected from the orbital venous plexus of mice on Days 0, 14, 28, 35, and 42 after immunization to detect humoral immune response levels. All mice were euthanized on Day 43 postimmunization.

### 4.14. Pig Immunization Experiment Plan

This experiment used 9‐week‐old crossbred Landrace pigs negative for FMDV antibodies to evaluate the humoral immune levels induced by various commercial inactivated FMDV vaccines and the 3D‐LS‐LOOP (T142P&Q153P) candidate vaccine. Pigs were randomly divided into 5 groups (*n* = 5 per group): FMDV Serotypes O/A Commercial Inactivated Vaccine A group; FMDV Serotypes O/A Commercial Inactivated Vaccine B group. FMDV Serotypes O Commercial Inactivated Vaccine C group, 3D‐LS‐LOOP (Wt) group, and 3D‐LS‐LOOP (T142P&Q153P) group. The immunization protocol is shown in Table S3. The 3D‐LS‐LOOP (T142P&Q153P) nanoparticle vaccine was prepared with reference to the method used in the previous mouse immunization experiment: the final concentration of the antigen was 150 μg/mL, and it was emulsified with Montanide ISA 201 VG adjuvant at a ratio of 1:1. All pigs were immunized via an intramuscular injection in the neck with 2 mL. The primary immunization was given on Day 0, and a booster immunization with the same dose was administered on Day 21. The serum was harvested from jugular vein blood samples collected at Day 0 and 40, and all pigs were humanely euthanized on Day 42.

### 4.15. Statistical Analysis

Statistical analyses were conducted using one‐way ANOVA, followed by Tukey’s multiple comparisons test with GraphPad Prism 8.0.1 software (GraphPad Software, Inc., San Diego, CA, USA). All experiments were performed in triplicate, and the exact *p* values are displayed.

## Author Contributions

Ping Qian, Xiangmin Li, and Jinyan Wu conceived the project, proofed the manuscript, and participated in the discussion and interpretation of the results. Nan Cao, Yamei Li, Xinghua Chen, Qiongqiong Zhao, Jinyan Zhang, Xianfei Shang, Junling Hou, Jinming Zhang, and Bo Yin performed the experiments. Nan Cao and Yamei Li analyzed the data and wrote the manuscript.

## Funding

This work was supported by grants from the National Key R&D Program of China (Grants 2021YFD1800300 and 2022YFD1800800), the National Natural Science Foundation of China (Grant 32072841), and the Huazhong Agricultural University Intelligent Research Institute of Food Health (Grant IRIFH202209) for their support of this research.

## Disclosure

All authors have read and approved the final manuscript.

## Conflicts of Interest

The authors declare no conflicts of interest.

## Supporting Information

Additional supporting information can be found online in the Supporting Information section.

## Supporting information


**Supporting Information** Figure S1: Construction of infectious clones of serotype O FMDV mutant strains based on site‐directed mutagenesis and homologous recombination. Figure S2: Restriction enzyme Not I mediated linearization of infectious clone plasmid for serotype O FMDV mutant. Table S1: Evaluation of FMD vaccines based on for C57BL /6 mice immunization. Table S2: Evaluation of the immune effect of FMD vaccines with mutations at amino acid sites 142 and 153 in the VP1 G–H loop in C57BL/6 mice. Table S3: Evaluation of swine immune effect of the different FMD vaccines.

## Data Availability

The data that support the findings of this study are openly available in Science DB at https://www.scidb.cn/datalist/index4, Reference Number 10.57760/sciencedb.37705.
