# Supplementary material for: Adaptive Evolution of Serotype O Foot‐and‐Mouth Disease Virus Under Vaccine Pressure: Combined VP1 T142/Q153 Mutations Drive Antigenic Alteration and Immune Evasion
Source: Transbound Emerg Dis. 2026 Jul 13;2026:4808637. doi: 10.1155/tbed/4808637 (PMC13358367; doi:10.1155/tbed/4808637)
Supplement: Supplementary file 1 — Supporting Information Figure S1: Construction of infectious clones of serotype O FMDV mutant strains based on site‐directed mutagenesis and homologous recombination. Figure S2: Restriction enzyme Not I mediated linearization of infectious clone plasmid for serotype O FMDV mutant. Table S1: Evaluation of FMD vaccines based on for C57BL /6 mice immunization. Table S2: Evaluation of the immune effect of FMD vaccines with mutations at amino acid sites 142 and 153 in the VP1 G–H loop in C57BL/6 mice. Table S3: Evaluation of swine immune effect of the different FMD vaccines. [file TBED-2026-4808637-s001.docx]

Adaptive evolution of serotype O Foot-and-Mouth Disease Virus under vaccine pressure: combined VP1 T142/Q153 mutations drive antigenic alteration and immune evasion

Nan Cao^1, 2, 4, 7^, Yamei Li ^1, 2, 4, 7^, Xinghua Chen ^1, 2, 4^, Qiongqiong Zhao ^1, 2, 4^, Jinyan Zhang ^1, 2, 4^, Xianfei Shang ^1, 2, 4^, Junling Hou ^5^, Jinming Zhang ^5^, Bo Yin ^6^, Jinyan Wu^5^ *, Xiangmin Li ^1, 2, 3, 4^ *, Ping Qian ^1, 2, 3, 4^*

^1^ National Key Laboratory of Agricultural Microbiology, Hubei Hongshan Laboratory, Huazhong Agricultural University, Wuhan, 430070, Hubei, China

^2^ College of Veterinary Medicine, Huazhong Agricultural University, Wuhan, 430070, Hubei, China

^3^ Hubei Jiangxia Laboratory, Wuhan, 430200, P. R. China.

^4^ Key Laboratory of Preventive Veterinary Medicine in Hubei Province, The Cooperative Innovation Center for Sustainable Pig Production, Wuhan, 430070, Hubei, China

^5^ State Key Laboratory for Animal Disease Control and Prevention, College of Veterinary Medicine, Lanzhou University, Lanzhou Veterinary Research Institute, Chinese Academy of Agricultural Sciences, Lanzhou, 730046, PR China

^6^ Shanghai Shen Lian Biomedical Corporation, 200241, Shanghai, China

^7^ These authors contribute equally to this work.

* Corresponding authors:

E-mail: qianp@mail.hzau.edu.cn (P. Qian); lixiangmin@mail.hzau.edu.cn (X. Li); wujinyan@caas.cn（J. Wu）

SUPPLEMENTARY FIGURES


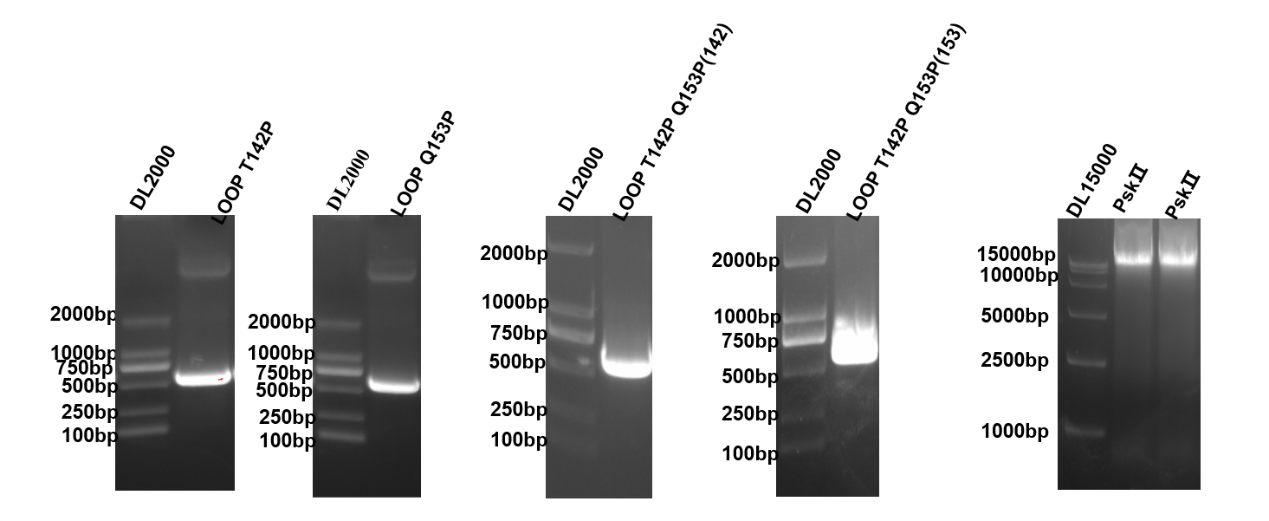


Supplementary figure 1 Construction of infectious clones of serotype O FMDV mutant strains based on site-directed mutagenesis and homologous recombination


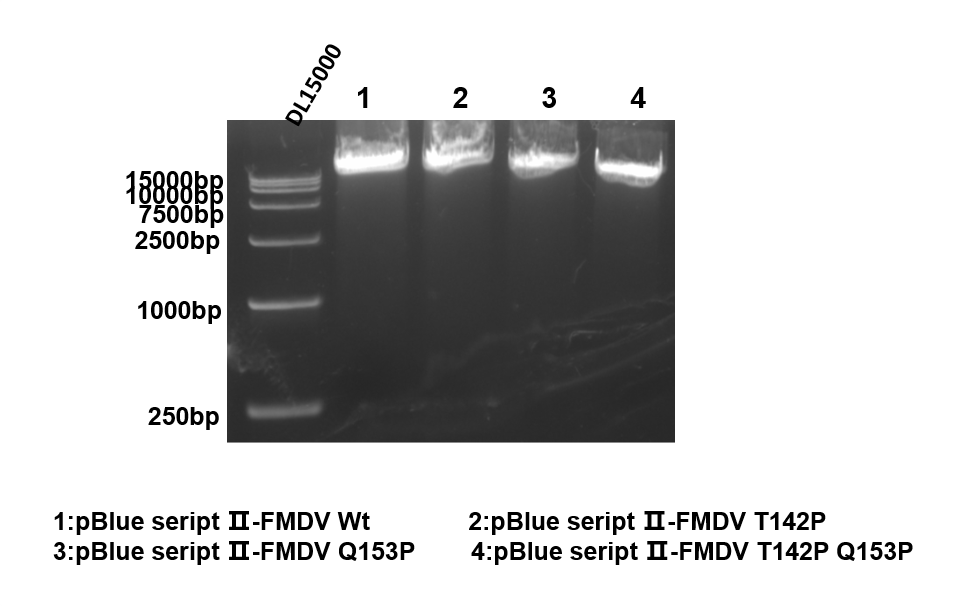


Supplementary figure 2 Restriction enzyme *Not I* mediated linearization of infectious clone plasmid for serotype O FMDV mutant

SUPPLEMENTARY TABLES

Supplementary table. 1 Evaluation of FMD vaccines based on for C57BL /6 mice immunization.

| Groups |  |  |  | Adjuvant | 0d | 21d |
| --- | --- | --- | --- | --- | --- | --- |
|  |  |  |  |  | Intramuscular injection(i.m.) | |
| 3D-LS-LOOP (Wt) |  |  |  | ISA 201 VG | 40μg/200μL | 40μg/200μL |
| 3D-LS-LOOP(T142P Q153P) |  |  |  | ISA 201 VG | 40μg/200μL | 40μg/200μL |

Supplementary table. 2 Evaluation of the immune effect of FMD vaccines with mutations at amino acid sites 142 and 153 in the VP1 G-H loop in C57BL/6 mice

| Groups |  |  |  | Adjuvant | 0d | 21d |
| --- | --- | --- | --- | --- | --- | --- |
|  |  |  |  |  | Intramuscular injection(i.m.) | |
| 3D-LS-LOOP(Wt) |  |  |  | ISA 201 VG | 40μg/200μL | 40μg/200μL |
| 3D-LS-LOOP(T142P) |  |  |  | ISA 201 VG | 40μg/200μL | 40μg/200μL |
| 3D-LS-LOOP(Q153P) |  |  |  | ISA 201 VG | 40μg/200μL | 40μg/200μL |
| 3D-LS-LOOP(T142P Q153P) |  |  |  | ISA 201 VG | 40μg/200μL | 40μg/200μL |

Supplementary table. 3 Evaluation of swine immune effect of the different FMD vaccines.

| Groups |  |  |  | Adjuvant | 0d | 21d |
| --- | --- | --- | --- | --- | --- | --- |
|  |  |  |  |  | Intramuscular injection(i.m.) | |
| inactivated vaccine A |  |  |  | ISA 201 VG | 2mL | 2mL |
| inactivated vaccine B |  |  |  | ISA 201 VG | 2mL | 2mL |
| inactivated vaccine C |  |  |  | ISA 201 VG | 2mL | 2mL |
| 3D-LS-LOOP(Wt) |  |  |  | ISA 201 VG | 300μg/2m L | 300μg/2mL |
| 3D-LS-LOOP(T142P Q153P) |  |  |  | ISA 201 VG | 300μg/2m L | 300μg/2mL |
